# Facile Synthesis of Novel CaIn_2_S_4_/ZnIn_2_S_4_ Composites with Efficient Performance for Photocatalytic Reduction of Cr(VI) under Simulated Sunlight Irradiation

**DOI:** 10.3390/nano8070472

**Published:** 2018-06-27

**Authors:** Siyu Xu, Jun Dai, Juan Yang, Jun You, Jingyi Hao

**Affiliations:** 1 Institute of Chemical Safety, School of Safety Science and Engineering, Henan Polytechnic University, Jiaozuo 454003, China; xusiyu2017@163.com (S.X.); daijun@hpu.edu.cn (J.D.); 2 The Collaborative Innovation Center of Coal Safety Production of Henan, Henan Polytechnic University, Jiaozuo 454003, China; 3 Institute of Applied Chemistry, College of Chemistry and Chemical Engineering, Henan Polytechnic University, Jiaozuo 454003, China; youjunhpu@163.com (J.Y.); haojingyi2018@163.com (J.H.)

**Keywords:** CaIn_2_S_4_/ZnIn_2_S_4_ composites, Cr(VI), photocatalysis, simulated sunlight, hydrothermal method

## Abstract

A series of novel and efficient heterostructured composites CaIn_2_S_4_/ZnIn_2_S_4_ have been synthesized using a facile hydrothermal method. XRD patterns indicate the as-prepared catalysts are two-phase composites of cubic phase CaIn_2_S_4_ and hexagonal phase ZnIn_2_S_4_. FESEM (field emission scanning electron microscope) images display that the synthesized composites are composed of flower-like microspheres with wide diameter distribution. UV–Vis diffuse reflectance spectra (DRS) show that the optical absorption edges of the CaIn_2_S_4_/ZnIn_2_S_4_ composites shift toward longer wavelengths with the increase of the CaIn_2_S_4_ component. The photocatalytic activities of the as-synthesized composites are investigated by using the aqueous-phase Cr(VI) reduction under simulated sunlight irradiation. This is the first report on the application of the CaIn_2_S_4_/ZnIn_2_S_4_ composites as stable and efficient photocatalysts for the Cr(VI) reduction. The fabricated CaIn_2_S_4_/ZnIn_2_S_4_ composites possess higher photocatalytic performance in comparison with pristine CaIn_2_S_4_ or ZnIn_2_S_4_. The CaIn_2_S_4_/ZnIn_2_S_4_ composite with a CaIn_2_S_4_ molar content of 30% exhibits the optimum photocatalytic activity. The primary reason for the significantly enhanced photoreduction activity is proved to be the substantially improved separation efficiency of photogenerated electrons/holes caused by forming the CaIn_2_S_4_/ZnIn_2_S_4_ heterostructured composites. The efficient charge separation can be evidenced by steady-state photoluminescence spectra (PLs) and transient photocurrent response. Based on the charge transfer between CaIn_2_S_4_ and ZnIn_2_S_4_, an enhancement mechanism of photocatalytic activity and stability for the Cr(VI) reduction is proposed.

## 1. Introduction

Semiconductor-based photocatalytic technology has exhibited great potential in green controlling of environmental contaminants and converting CO_2_ to valuable chemicals [1,2,3,4,5,6]. However, the most studied photocatalysts (such as TiO_2_, ZnO, and ZnS) are active only under ultraviolet light irradiation. In view of the practical applications, visible light active photocatalysts with narrow bandgaps are greatly desirable. A great deal of research and efforts have been devoted to fabricating and synthesizing visible-light-driven catalysts, [7,8,9,10]. For instance, visible-light photocatalysts based on metal sulfide, including the doped or functionalized ZnS [7,8], CdIn_2_S_4_ [9], ZnIn_2_S_4_ [10,11], CdIn_2_S_4_/ZnIn_2_S_4_ [12], and Cu_2_ZnSnS_4_ [13] have received broad attention in recent decades.

Among these metal sulfides, ZnIn_2_S_4_, as a ternary chalcogenide semiconductor possessing a narrow bandgap (2.34–2.48 eV), has aroused great interest in the field of visible-light-driven contaminants’ degradation and hydrogen production by water splitting [10,11]. Although the studies have revealed that ZnIn_2_S_4_ has strong visible-light absorption, the catalytic activity of pristine ZnIn_2_S_4_ is lower than expected due to fast recombination of photogenerated charge carriers. Many efforts have been made to solve these drawbacks, including morphologies control, metal doping, and heterostructured composites construction [14,15,16,17]. The fabrication of heterojunction catalysts by coupling two kinds of semiconductor particles with a suitable energy band structure is identified as a valid method to promote photocatalytic activity because of the resulting effective separation of the photoinduced charge [18,19]. To improve the photocatalytic activity of pure ZnIn_2_S_4_, the heterojunction photocatalysts have been reported by hybridization with TiO_2_, CdS, and C_3_N_4_ [20,21,22].

On the other hand, the metal sulfide semiconductors with narrow bandgaps generally undergo photocorrosion induced by the oxidative process of photogenerated holes, which result in poor photostability or deactivation during long-term recycling use under sunlight irradiation. For instance, CdS particles possess excellent catalytic activity, acceptable stability, and photocorrosion resistance under visible-light illumination, whereas the catalyst becomes unstable when exposed to sunlight irradiation [23]. As a visible-light photocatalyst, ZnIn_2_S_4_ also suffers from the critical drawback of high photocorrosion under sunlight illumination [24]. Very recently, Zhao et al. have synthesized chemically cross-linked ZnIn_2_S_4_/RGO heterostructured catalysts and investigated the sunlight-driven photocatalytic performance of 4-nitrophenol degradation. The experimental results demonstrate the as-fabricated ZnIn_2_S_4_/RGO possessed not only the enhanced visible-light catalytic performance but also prominently improved sunlight stability [24]. Therefore, to practically utilize sunlight-driven photocatalytic technology to control contaminants, the employed catalysts must be not only visible-light active but also stable when subjected to sunlight illumination.

CaIn_2_S_4_ is also an attractive ternary chalcogenide with a narrow bandgap (~1.9 eV) and meanwhile, is the cheapest alkaline earth metal-based semiconductor, which can serve as a potential visible-light catalyst. However, only a few of the investigations on the construction and synthesis of CaIn_2_S_4_-based composite photocatalysts have been reported to date [25,26,27]. Similar to other narrow-bandgap catalysts, the photocatalytic degradation efficiency of CaIn_2_S_4_ alone is very limited owing to the poor separation efficiency of photogenerated electrons and holes. By constructing the composite catalysts, such as CaIn_2_S_4_-RGO, CaIn_2_S_4_/g-C_3_N_4_, and CaIn_2_S_4_/TiO_2_, the photocatalytic activity of CaIn_2_S_4_ has been significantly improved. For instance, direct Z-scheme CaIn_2_S_4_/TiO_2_ catalysts with different CaIn_2_S_4_ weight percentages were prepared, and the photocatalytic performance of these composite catalysts was investigated by the degradation of isoniazid and metronidazole in the pharmaceutical wastewater. The improved catalytic performance can be ascribed to the significantly suppressed recombination of photoinduced charge carriers based on the Z-scheme charge transfer over CaIn_2_S_4_/TiO_2_ catalysts. The aforementioned CaIn_2_S_4_-based composites show higher photocatalytic performance than the pure CaIn_2_S_4_ or TiO_2_; nevertheless, the preparation processes are relatively complicated, and these composites cannot be obtained through one-step synthesis. Further research on the synthesis of the CaIn_2_S_4_-based composite photocatalysts by one-step hydrothermal methods is highly necessary to improve the photocatalytic activity of CaIn_2_S_4_.

Hexavalent Cr(VI) ions have been listed as one of the priority pollutants by the United States Environmental Protection Agency (US EPA) [28], due to the high toxicity, mutagenicity, and teratogenicity to the aquatic environment and human beings. The elimination of Cr(VI) ions have received increasing attention in the field of wastewater purification. The traditional techniques for removing Cr(VI) ions generally include precipitation, adsorption, ion-exchange, electro-coagulation [29], membrane separation, and photocatalytic reduction [30,31]. Considering solar energy conversion, photocatalytic reduction is postulated to be an efficient and green technology for the elimination of Cr(VI) ions from contaminated water. The highly toxic Cr(VI) can be photoreduced to less harmful Cr(III) by means of a certain photocatalyst and reaction system.

In this study, flower-like CaIn_2_S_4_/ZnIn_2_S_4_ heterojunction composites are successfully synthesized by using a one-step hydrothermal method, and the photocatalytic performance of the Cr(VI) reduction is investigated under simulated sunlight illumination. To our knowledge, this is the first report about the CaIn_2_S_4_/ZnIn_2_S_4_ heterojunction composites for the photocatalytic reduction of Cr(VI). The heterojunction composites exhibit much higher reduction efficiency of Cr(VI) than the pure CaIn_2_S_4_ or ZnIn_2_S_4_. Meanwhile, the CaIn_2_S_4_/ZnIn_2_S_4_ composites present excellent solar stability, and the photocorrosion of ZnIn_2_S_4_ is dramatically inhibited by constructing the CaIn_2_S_4_/ZnIn_2_S_4_ composites. The detailed mechanism of enhanced photocatalytic performance for the Cr(VI) reduction over the CaIn_2_S_4_/ZnIn_2_S_4_ composites is also proposed.

## 2. Materials and Methods

### 2.1. Materials

All the chemicals were of analytical grade and used as received without further purification. Calcium nitrate (Ca(NO_3_)_2_), zinc sulfate (ZnSO_4_), indium chloride (InCl_3_), thioacetamide (TAA), potassium dichromate, sulfuric acid (H_2_SO_4_), and ammonium oxalate were obtained from Aladdin Industrial Inc. (Shanghai, China). Diphenylcarbazide (DPC) was purchased from J & K Scientific Ltd. (Beijing, China). Double distilled water was used throughout this study.

### 2.2. Synthesis of Composite Photocatalysts

The CaIn_2_S_4_/ZnIn_2_S_4_ composite photocatalysts were prepared by hydrothermal route. Taking 20% CaIn_2_S_4_/ZnIn_2_S_4_ as an example, in a typical synthesis process, 0.2 mmol Ca(NO_3_)_3_·4H_2_O and 0.8 mmol ZnSO_4_·7H_2_O were added into 40 mL of distilled water, followed by vigorous stirring for 30 min to form a clear solution. After that, 2 mmol InCl_3_·4H_2_O and 8 mmol TAA were put into the above-obtained mixed solution and stirred for additional 60 min at room temperature. The reaction mixture was then transferred into a Teflon-lined steel autoclave (Zhengxin Instrument, Yancheng, China), which was heated at 180 °C for 12 h. Finally, the obtained yellow solid was collected by filtration, washed with distilled water several times, and dried at 60 °C for 8 h. On that basis, different CaIn_2_S_4_/ZnIn_2_S_4_ composites with CaIn_2_S_4_ molar ratios of 5%, 10%, 20%, 30%, and 50% were synthesized and denoted as 5% CaIn_2_S_4_/ZnIn_2_S_4_, 10% CaIn_2_S_4_/ZnIn_2_S_4_, 20% CaIn_2_S_4_/ZnIn_2_S_4_, 30% CaIn_2_S_4_/ZnIn_2_S_4_, and 50% CaIn_2_S_4_/ZnIn_2_S_4_, respectively.

For comparison, the pure ZnIn_2_S_4_ was prepared by using a similar process without Ca(NO_3_)_3_. 1.0 mmol ZnSO_4_·7H_2_O was put into 40 mL of distilled water and stirred for 30 min. Then, 2 mmol InCl_3_·4H_2_O and 8 mmol TAA were added to the above-obtained solution and stirred for another 60 min. This mixture was subsequently transferred into a Teflon-lined steel autoclave and heated at 180 °C for 12 h. The precipitate was finally washed with deionized water and dried at 60 °C for 8 h to obtain pure ZnIn_2_S_4_. For the preparation of the pure CaIn_2_S_4_, 1.0 mmol Ca(NO_3_)_3_·4H_2_O was added into 40 mL of distilled water and stirred for 30 min. After that, 2 mmol InCl_3_·4H_2_O and 8 mmol TAA were put into the above solution and then stirred for another 60 min. The obtained mixture was transferred into a Teflon-lined steel autoclave and heated at 180 °C for 12 h. The orange precipitate was finally washed with deionized water and dried at 60 °C for 8 h to obtain pure CaIn_2_S_4_.

### 2.3. Material Characterization

The crystal structures of the as-prepared composites were investigated by using an X-ray diffractometer (XRD, Bruker D8 Advance, Karlsruhe, Germany), with Cu Kα radiation (λ = 0.15405 nm). The morphologies of the synthesized samples were observed by a field emission scanning electron microscope (FEI, Quanta250, Hillsboro, OR, USA). The X-ray photoelectron spectroscopy (XPS) experiment was carried out by using a Thermo Scientific ESCALAB 250xi system (Waltham, MA, USA), equipped with an Al anode. The BET (Brunauer-Emmett-Teller) surface areas of the composite samples were determined on a surface area analyzer (Autosorb-IQ, Quantachrome, Boynton Beach, FL, USA). UV–visible diffuse reflectance spectra (DRS) were collected on a Shimadzu UV-2550 spectrometer (Kyoto, Japan) using BaSO_4_ as the reflectance standard. Photoluminescence spectra (PLs) were measured by using an Edinburgh FLS 980 fluorescence spectrometer (Livingston, UK), with a 330 nm excitation wavelength. The transient photocurrent measurement was performed on a CHI electrochemical workstation (CHI 760D, Shanghai, China) in the standard three-electrode system. Ag/AgCl electrode and a platinum wire were employed as the reference electrode and the counter electrode, respectively. The working electrodes were prepared according to our previous report [32]. The photocurrent was measured in the electrolyte of a 0.5 mol/L Na_2_SO_4_ aqueous solution (pH ~6.8) at a bias of +0.5 V. The light source was the same as that used in the photocatalytic experiments.

### 2.4. Photocatalytic Reduction of Cr(VI)

For the photocatalytic reduction of Cr(VI) under simulated sunlight irradiation, a 300 W xenon lamp (PLS-SXE 300, Perfect Light Co. Ltd., Beijing, China) was used as the light source. The incident light intensity was 75 mW/cm^2^. In the typical photocatalytic test, 50 mg of photocatalyst was suspended in 50 mL of 20 mg/L Cr(VI) aqueous solution. After adding 5 mg of ammonium oxalate (a scavenger for photo-hole), the suspension was magnetically stirred in the dark for 30 min to establish an adsorption–desorption equilibrium. As the photocatalytic reduction proceeded, 3 mL of the reaction solution was taken out at a given time interval and centrifuged to remove the catalyst particles. The concentrations of Cr(VI) were determined colorimetrically at 540 nm using the diphenylcarbazide (DPC) method on a Shimadzu UV-160A UV–Vis spectrophotometer [33]. In addition, the total Cr ions concentrations were measured by an inductively coupled plasma-optical emission spectrophotometer (Agilent 725 ICP-OES, Palo Alto, CA, USA).

The apparent quantum efficiency (AQE) of Cr(VI) the photocatalytic reduction was measured under the same photocatalytic reaction conditions, except by using a 420 nm bandpass filter. The apparent quantum efficiencies were calculated according to the following equation:

$$\mathrm{AQE}\left(\%\right)=\frac{\mathrm{Number}\;\mathrm{of}\;\mathrm{reacted}\;\mathrm{electrons}}{\mathrm{Number}\;\mathrm{of}\;\mathrm{incident}\;\mathrm{photons}}\times 100\;=\frac{3\times \mathrm{number}\;\mathrm{of}\;\mathrm{reduced}\;\mathrm{Cr}\left(\mathrm{VI}\right)\;}{\mathrm{Number}\;\mathrm{of}\;\mathrm{incident}\;\mathrm{photons}}\times 100$$

$$\mathrm{Number}\;\mathrm{of}\;\mathrm{incident}\;\mathrm{photons}=\;\frac{I\times A\times t}{E\left(\lambda =420\;\mathrm{nm}\right)}\;E\left(\lambda =420\;\mathrm{nm}\right)=\frac{h\times c}{\lambda }$$

where *I*, *A*, *t*, *c*, *λ*, and *h* are light intensity, light exposure area, irradiation time, light velocity, light wavelength, and Planck constant, respectively.

To evaluate the catalytic stability of the CaIn_2_S_4_/ZnIn_2_S_4_ composites, the photocatalysts—after the first run for the Cr(VI) reduction—were separated by centrifugation from the suspension and washed with 1 M nitrite acid solution and deionized water. After being dried at 60 °C, the recovered photocatalysts were reused for the next run of the photocatalytic Cr(VI) reductions under the same experimental conditions.

## 3. Results and Discussion

### 3.1. XRD Analysis and BET Surface Area

**Figure 1:**
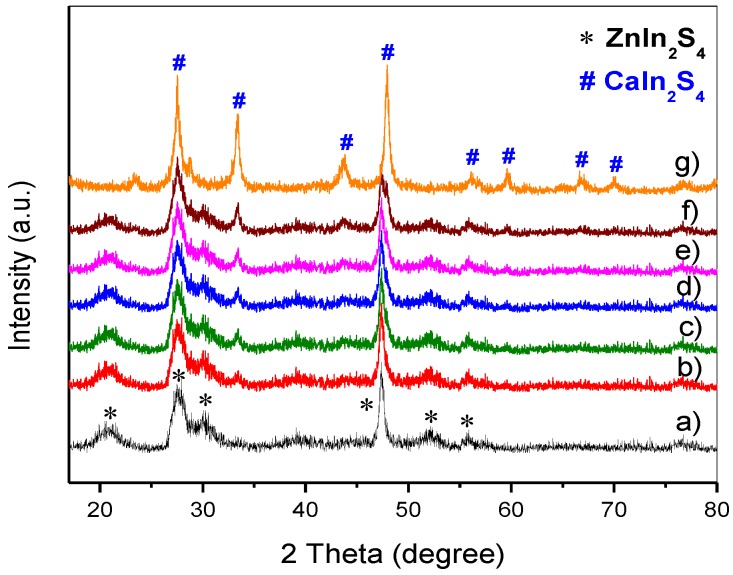
XRD patterns of pure ZnIn_2_S_4_, CaIn_2_S_4_, and CaIn_2_S_4_/ZnIn_2_S_4_ composites: (**a**) pure ZnIn_2_S_4_, (**b**) 5% CaIn_2_S_4_/ZnIn_2_S_4_, (**c**) 10% CaIn_2_S_4_/ZnIn_2_S_4_, (**d**) 20% CaIn_2_S_4_/ZnIn_2_S_4_, (**e**) 30% CaIn_2_S_4_/ZnIn_2_S_4_, (**f**) 50% CaIn_2_S_4_/ZnIn_2_S_4_, and (**g**) pure CaIn_2_S_4_.

The phase composition and crystalline properties of the CaIn_2_S_4_/ZnIn_2_S_4_ composite samples were analyzed by XRD. Figure 1 displays the XRD patterns of the pure ZnIn_2_S_4_, CaIn_2_S_4_, and CaIn_2_S_4_/ZnIn_2_S_4_ composites. As indicated in Figure 1, the diffraction peaks of the pure ZnIn_2_S_4_ at 2θ = 21.1°, 27.6°, 30.3°, 47.4°, 52.0°, and 55.6° corresponded to (006), (102), (104), (110), (116), and (200) crystal planes of hexagonal phase ZnIn_2_S_4_ (JCPDS NO. 65-2023), respectively [24]. No other diffraction peaks were detected in the XRD pattern of ZnIn_2_S_4_, indicating that the obtained ZnIn_2_S_4_ is highly pure. With the addition of Ca(NO_3_)_3_ in the preparation process, new diffraction peaks appeared in the XRD patterns, which belonged to the characteristic peaks of the cubic phase CaIn_2_S_4_ (JCPDS No. 16-0341) [25,26,27]. It can be also observed from Figure 1 that the intensity of the diffraction peaks belonging to the cubic CaIn_2_S_4_ phase increased gradually by increasing the mole proportion of CaIn_2_S_4_, which indicated the existence of both CaIn_2_S_4_ and ZnIn_2_S_4_ in the as-synthesized composites. In addition, the diffraction peaks corresponding to binary sulfides, oxides, and other new compounds were not observed, indicating that CaIn_2_S_4_ and ZnIn_2_S_4_ maintained the pure phase and no impurities were formed in the obtained CaIn_2_S_4_/ZnIn_2_S_4_ composites.

The BET surface area of the pure ZnIn_2_S_4_, CaIn_2_S_4_, and CaIn_2_S_4_/ZnIn_2_S_4_ composites was measured, and the results are summarized in Table 1. As can be noted from Table 1, the specific surface area of the CaIn_2_S_4_/ZnIn_2_S_4_ composites decreased slightly with the continuous increment of CaIn_2_S_4_ component. The actual molar ratios of Ca–Zn in the CaIn_2_S_4_/ZnIn_2_S_4_ composites were determined by inductively coupled plasma elemental analysis, as presented in Table 1. It can be observed from Table 1 that the experimentally measured molar ratios of Ca–Zn in the synthesized composites were close to those of the added proportions in the preparation process.

### 3.2. SEM and Elemental Mapping Analysis

**Figure 2:**
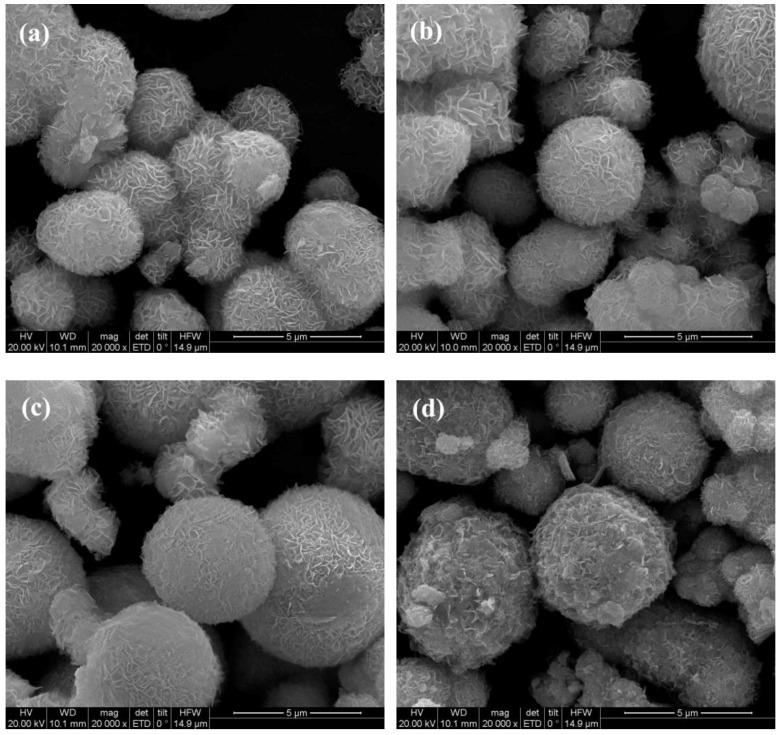
FE-SEM (field emission scanning electron microscope) images of (**a**) pure ZnIn_2_S_4_, (**b**) 10% CaIn_2_S_4_/ZnIn_2_S_4_, (**c**) 30% CaIn_2_S_4_/ZnIn_2_S_4_, and (**d**) pure CaIn_2_S_4_.

**Figure 3:**
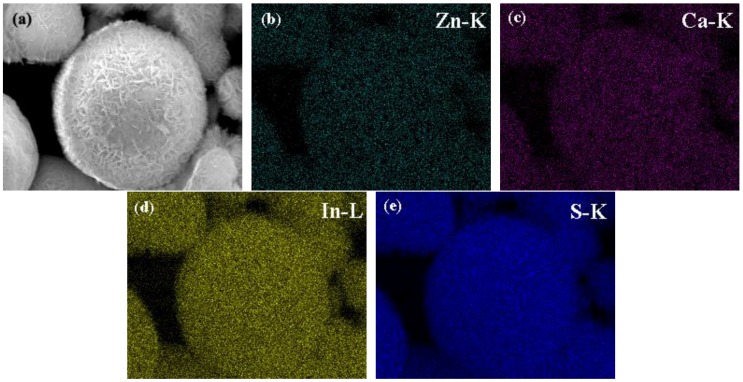
The EDS (Energy Dispersive Spectroscopy) elemental mapping images of the 30% CaIn_2_S_4_/ZnIn_2_S_4_ sample: (**a**) SEM image, (**b**) Zn-K, (**c**) Ca-K, (**d**) In-L, and (**e**) S-K, respectively.

The SEM images of the as-synthesized pure ZnIn_2_S_4_, CaIn_2_S_4_, and CaIn_2_S_4_/ZnIn_2_S_4_ composites are indicated in Figure 2. As can be observed from Figure 2, the pure ZnIn_2_S_4_ was composed of hierarchical microspheres with a wide distribution of diameter, which was consistent with the previous reports [17,24]. Introducing the Ca component had almost no influence on the morphologies of the CaIn_2_S_4_/ZnIn_2_S_4_ composites, which also exhibited flower-like microspheres constructed by numerous nanosheets in the form of random self-assembly. As a consequence, the porous structures with wide pore-size distribution could be expected. This would benefit the photocatalytic reaction by increasing the specific surface area. Moreover, the formation of the CaIn_2_S_4_/ZnIn_2_S_4_ heterojunction was evidenced by the elemental mapping of the as-synthesized composites (Figure 3). Maps of Zn–K, Ca–K, In–L, and S–K display the same shape and location, indicating the coexistence of ZnIn_2_S_4_ and CaIn_2_S_4_ components in the obtained composites. This provided solid evidence for the formation of CaIn_2_S_4_/ZnIn_2_S_4_ heterostructured composites.

### 3.3. XPS Analysis and Optical Properties

**Figure 4:**
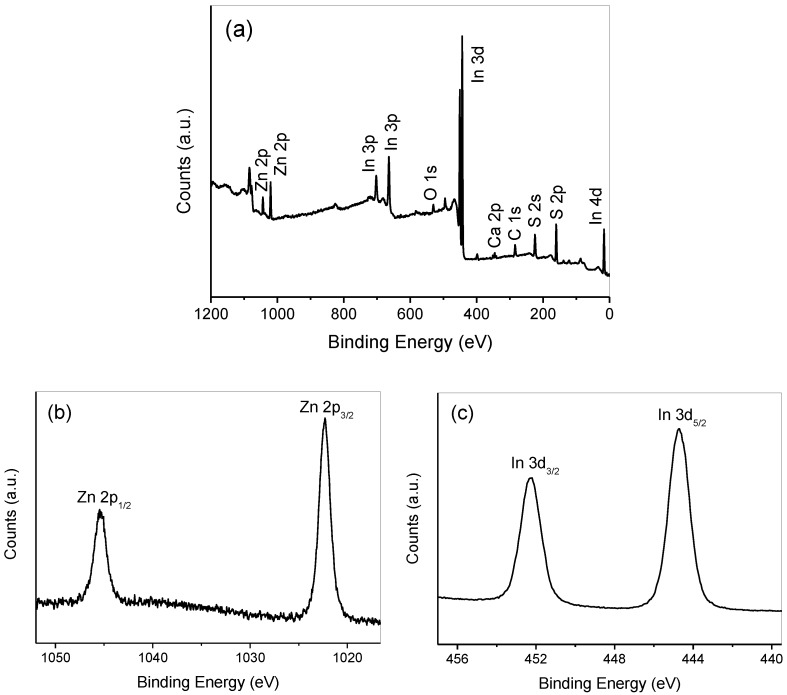

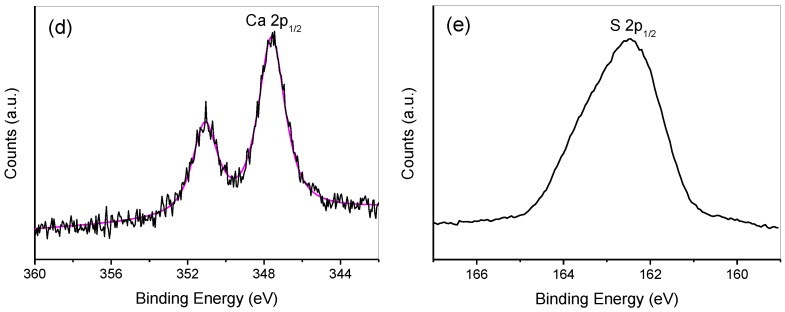
Typical XPS survey spectrum of 30% CaIn_2_S_4_/ZnIn_2_S_4_ (**a**), high-resolution XPS spectra of Zn 2p (**b**), In 3d (**c**), Ca 2p (**d**), and S 2p (**e**), respectively.

To determine the elemental composition and the corresponding chemical states of the synthesized composites, the XPS spectra of the 30% CaIn_2_S_4_/ZnIn_2_S_4_ sample are indicated in Figure 4. As can be seen from Figure 4a, the survey spectrum indicated the presence of Zn, Ca, In, and S elements in the 30% CaIn_2_S_4_/ZnIn_2_S_4_ sample. The high-resolution XPS spectrum for Zn is presented in Figure 4b. Two characteristic XPS signals were observed at binding energies of 1022.4 and 1045.3 eV, which were ascribed to Zn^2+^ 2p_1/2_ and Zn^2+^ 2p_3/2_, respectively [33,34]. The high-resolution XPS spectrum of In 3d is displayed in Figure 4c. The characteristic peaks centered at 444.7 and 452.3 eV can be attributed to the In 3d_3/2_ and In 3d_5/2_ signals of In^3+^ species, respectively [27,34]. In Figure 4d, the two peaks at the binding energies of 351.1 and 347.6 eV corresponded to the 2p_3/2_ and 2p_1/2_ levels of Ca^2+^. Figure 4e shows an XPS signal centered at 162.5 eV, which can be assigned to the 2p_1/2_ level of S^2−^ in the as-prepared CaIn_2_S_4_/ZnIn_2_S_4_ composites [34]. These results further indicate that CaIn_2_S_4_/ZnIn_2_S_4_ composites can be successfully synthesized through a one-step hydrothermal reaction process.

**Figure 5:**
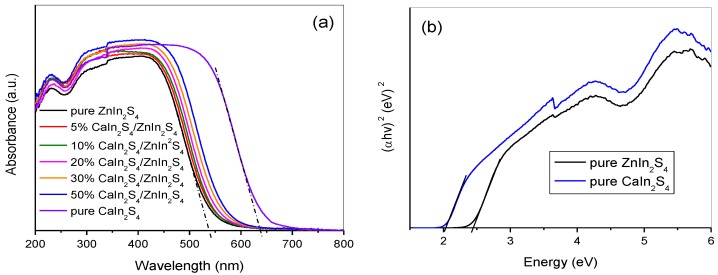
(**a**) UV–Vis DRS of the pure ZnIn_2_S_4_, CaIn_2_S_4_, and CaIn_2_S_4_/ZnIn_2_S_4_ composites, (**b**) Plots of (α*hv*)^2^ vs. light energy (*hv*) of the pure ZnIn_2_S_4_ and CaIn_2_S_4_.

The optical absorption properties of the as-obtained CaIn_2_S_4_/ZnIn_2_S_4_ composites were analyzed by UV–Vis DRS, and the results are depicted in Figure 5a. The absorption edges of the pure ZnIn_2_S_4_ and CaIn_2_S_4_ samples were at around 537 nm and 638 nm, respectively. As can be also noted from Figure 5a, the absorption edges of the CaIn_2_S_4_/ZnIn_2_S_4_ samples were gradually red-shifted from 537 to 570 nm as the molar percentage of the CaIn_2_S_4_ component increased to 50%. The photoresponse of the CaIn_2_S_4_/ZnIn_2_S_4_ composites in visible-light region was significantly improved by comparison with that of the pure ZnIn_2_S_4_. Moreover, the UV–Vis DRS shown in Figure 5a were all very steep, demonstrating the visible-light absorption was ascribed to the intrinsic band transition instead of the transition from impurity levels [35]. Based on the optical absorption theory of the bandgap semiconductor, the bandgap energy of the pure CaIn_2_S_4_ and ZnIn_2_S_4_ can be calculated by the Equation (1) [36] as follows:

α*hv* = *A* (*hv* − *E*_g_)*^n^*^/2^ (1)

where α, *v*, *E*_g_, and *A* represent the absorption coefficient, light frequency, bandgap energy, and a constant, respectively. The value of *n* depends on the type of optical transition of a semiconductor (*n* = 1 for the direct transition and *n* = 4 for the indirect transition). According to the previous literature [27,37], ZnIn_2_S_4_ and CaIn_2_S_4_ are direct-transition semiconductors, and thus, the bandgap energies of CaIn_2_S_4_ and ZnIn_2_S_4_ can be estimated from the plots of (α*hv*)^2^ versus light energy (*hv*). As illustrated in Figure 5b, the estimated bandgaps were 2.03 and 2.43 eV for the pure CaIn_2_S_4_ and ZnIn_2_S_4_, respectively, which matched well with the literature values [15,27]. For the composite catalysts, the photocatalytic performance was primarily determined by the valence band (VB) and conduction band (CB) energy levels of the constituent semiconductors. Based on the following equations, the VB and CB positions of the pristine ZnIn_2_S_4_ and CaIn_2_S_4_ can be obtained:

*E*_VB_ = *χ* − *E*_e_ + 0.5 *E*_g_ (2)

*E*_CB_ = *E*_VB_ − *E*_g_ (3)

where *E*_VB_ and *E*_CB_ are the potential of the VB and CB edge, *E*_g_ is the bandgap energy, χ is the geometric mean of the absolute electronegativity of the constituent atoms in the semiconductor, and *E*_e_ is the energy of free electrons on the hydrogen scale with a value of 4.5 eV. The χ values of ZnIn_2_S_4_ and CaIn_2_S_4_ were 4.86 and 4.39 eV, respectively. Based on the above empirical equations, the *E*_VB_ values of ZnIn_2_S_4_ and CaIn_2_S_4_ were estimated to be +1.58 and +0.91 eV. The corresponding *E*_CB_ values were also calculated to be −0.85 and −1.12 eV, respectively.

### 3.4. Photocatalytic Activity

**Figure 6:**
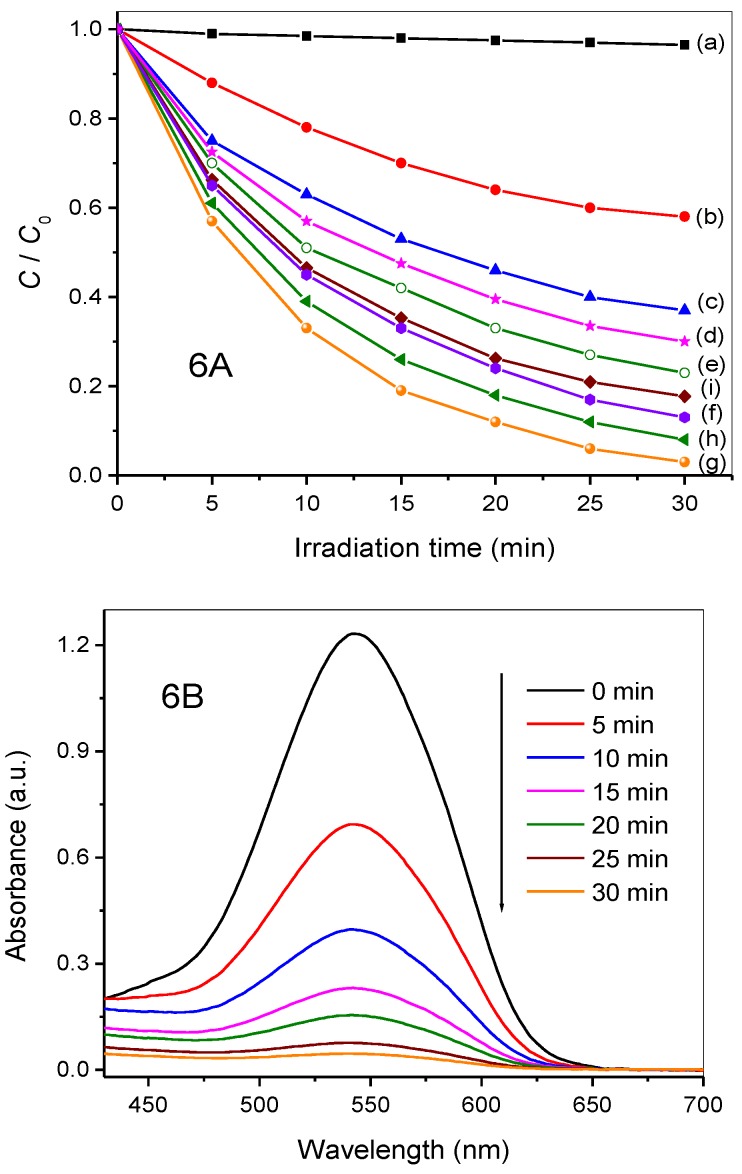

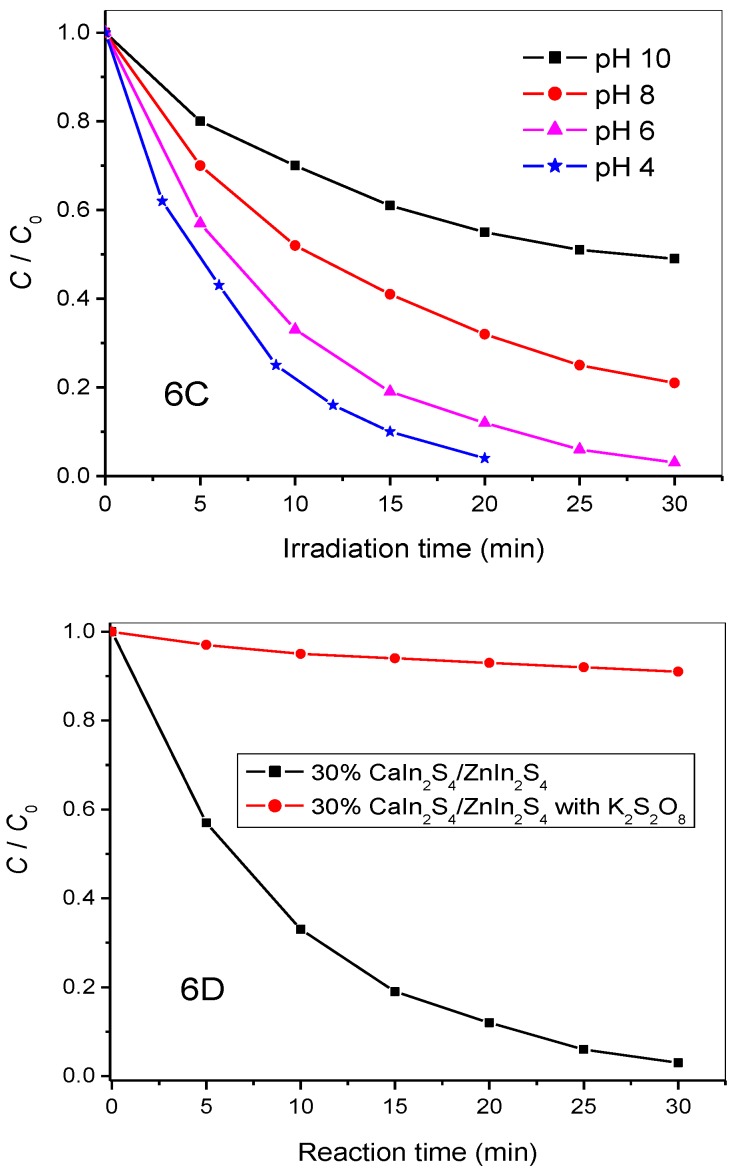
(**A**) Photocatalytic reduction of Cr(VI) as a function of irradiation time over different catalysts: (a) no catalyst, (b) pure CaIn_2_S_4_, (c) pure ZnIn_2_S_4_, (d) 5% CaIn_2_S_4_/ZnIn_2_S_4_, (e) 10% CaIn_2_S_4_/ZnIn_2_S_4_, (f) 20% CaIn_2_S_4_/ZnIn_2_S_4_, (g) 30% CaIn_2_S_4_/ZnIn_2_S_4_, (h) 50% CaIn_2_S_4_/ZnIn_2_S_4_, and (i) mechanically mixed 30%CaIn_2_S_4_ + 70%ZnIn_2_S_4_; (**B**) time-dependent absorption spectral pattern of diphenylcarbazide (DPC)-Cr(VI) complex solutions after the reduction over 30% CaIn_2_S_4_/ZnIn_2_S_4_ (pH = 6); (**C**) photocatalytic reduction of Cr(VI) under different pH values over 30% CaIn_2_S_4_/ZnIn_2_S_4_; (**D**) the controlled experiment for photocatalytic reduction of Cr(VI) over the 30% CaIn_2_S_4_/ZnIn_2_S_4_ composites with the addition of K_2_S_2_O_8_ (0.1 mmol) as a scavenger for photoinduced electrons.

The photocatalytic performances of the ZnIn_2_S_4_, CaIn_2_S_4_, and CaIn_2_S_4_/ZnIn_2_S_4_ composites were investigated by the aqueous-phase Cr(VI) reduction under simulated sunlight irradiation (Figure 6A). Cr(VI) cannot be reduced in the absence of light illumination or photocatalysts. The pure ZnIn_2_S_4_ displayed a relatively higher photocatalytic activity of the Cr(VI) reduction than the CaIn_2_S_4_ sample. About 42% of Cr(VI) was reduced over the pure CaIn_2_S_4_, while ZnIn_2_S_4_ can reduce 63% of Cr(VI) after irradiation of 30 min. All the CaIn_2_S_4_/ZnIn_2_S_4_ composites exhibited higher photocatalytic efficiency than the pristine CaIn_2_S_4_ and ZnIn_2_S_4_, indicating that the combination of CaIn_2_S_4_ and ZnIn_2_S_4_ can improve the photocatalytic reduction performance of these composites.

For the synthesized CaIn_2_S_4_/ZnIn_2_S_4_ composite catalysts, the photocatalytic were closely associated with the contents of the CaIn_2_S_4_ component. As can be noted from Figure 6A, the photocatalytic activities of the CaIn_2_S_4_/ZnIn_2_S_4_ composite catalysts increased with the increment of component CaIn_2_S_4_. The 30% CaIn_2_S_4_/ZnIn_2_S_4_ composite photocatalyst exhibited the highest activity for the Cr(VI) reduction, whereas the greater increase in the amount of CaIn_2_S_4_ resulted in a decrease in the Cr(VI) reduction rates. It can be ascribed to the low photocatalytic activity of the pure CaIn_2_S_4_ because of the slow separation of the photogenerated charge carriers. Moreover, the apparent quantum efficiencies (AQE) of the Cr(VI) photocatalytic reduction over the synthesized composites were also calculated, and the corresponding results are summarized in Table 1. As indicated in Table 1, the AQE of the 30% CaIn_2_S_4_/ZnIn_2_S_4_ nanocomposite catalyst reached 6.6%, which presented higher than that of the pure ZnIn_2_S_4_ (3.7%) or pure CaIn_2_S_4_ (2.3%). In addition, the AQE for the Cr(VI) reduction increased gradually with the increase of the CaIn_2_S_4_ constituent when the addition ratio of the CaIn_2_S_4_ precursor was no more than 30%. However, further increasing the molar ratio of CaIn_2_S_4_ led to a decrease in the AQE. This suggests that the heterostructured composites containing a suitable amount of CaIn_2_S_4_ and ZnIn_2_S_4_ contributed to improving optimally the photoactivity for the Cr(VI) reduction. Therefore, the optimal 30% CaIn_2_S_4_/ZnIn_2_S_4_ has been chosen as a representative catalyst for the following studies.

To further clarify the influence of the heterostructure on the photocatalytic performance of the Cr(VI) reduction, the 30% CaIn_2_S_4_/ZnIn_2_S_4_ sample was compared to its mechanical mixing counterpart sample 30% CaIn_2_S_4_ + 70% ZnIn_2_S_4_. The photocatalytic activity of the Cr(VI) reduction over the physical mixture was much lower than that of the 30% CaIn_2_S_4_/ZnIn_2_S_4_ composite obtained via the one-step hydrothermal method. This result shows that the heterojunction formed between the CaIn_2_S_4_ and ZnIn_2_S_4_ contributed to improving photocatalytic efficiency of the Cr(VI) reduction.

When using the optimal 30% CaIn_2_S_4_/ZnIn_2_S_4_ as a photocatalyst, the change in the temporal absorption spectra of the DPC-Cr(VI) complex solution is illustrated in Figure 6B. The absorption peak at 540 nm belonging to the DPC-Cr(VI) complex decreased rapidly with the increase of light irradiation time, and it almost vanished after light illumination for 30 min. To gain more insight into the photocatalytic process, the total Cr ions concentrations over the 30% CaIn_2_S_4_/ZnIn_2_S_4_ photocatalyst after the treated samples were measured by ICP emission spectrometer (Figure S1, Supplementary Materials). As indicated in Figure S1, the initial concentration of Cr(VI) is 19.6 ppm. The measured total Cr ions concentration after 30 min light irradiation were found to be 19.1 ppm. This result demonstrates that almost no Cr(0) was produced in the present photocatalytic system. That is to say, Cr(VI) was primarily reduced to Cr(III) by the photogenerated electrons of the CaIn_2_S_4_/ZnIn_2_S_4_ composites.

In addition, the photocatalytic experiments for the Cr(VI) reduction under different pH conditions with 30% CaIn_2_S_4_/ZnIn_2_S_4_ were also carried out, and the corresponding results are presented in Figure 6C. In a photocatalytic system, the reduction efficiency of the aqueous Cr(VI) was greatly influenced by the pH value according to the previous reports [30]. Cr(VI) existed in two forms in alkaline and acid medium, respectively. CrO_4_^2−^ is predominant in the alkaline medium, whereas Cr_2_O_7_^2−^ plays a major role in the acid medium. The chemical redox reaction can be outlined as follows:

CrO_4_^2−^ + 4H_2_O + 3e^−^ → Cr(OH)_3_↓ + 5OH^−^ (alkaline) (4)

14H^+^ + Cr_2_O_7_^2−^ + 6e^−^ → 2Cr^3+^ + 7H_2_O (acid) (5)

As depicted in Figure 6C, the reduction rate of Cr(VI) increased with the decrease of the pH value. About 38.7%, 58.8%, 80.6%, and 89.0% of Cr(VI) are reduced at pH 10, 8, 6, and 4 in the first 15 min, respectively. This could be due to the fact that the Cr(OH)_3_ precipitate produced in the alkaline medium covers the activity sites of the photocatalysts [38]. The results demonstrate the acidic condition was more beneficial to the photocatalytic reduction of Cr(VI) over the synthesized CaIn_2_S_4_/ZnIn_2_S_4_ composites.

Additionally, the controlled experiment by adding K_2_S_2_O_8_ (the trapping agent of photo-generated electrons, 0.1 mmol) [39] into the photocatalytic system of the Cr(VI) reduction was performed, and the corresponding result is depicted in Figure 6D. It can be seen clearly that the Cr(VI) reduction over the optimal photocatalyst 30% CaIn_2_S_4_/ZnIn_2_S_4_ hardly occurs in the presence of K_2_S_2_O_8_, which indicates that the reduction of Cr(VI) is conducted by photogenerated electrons under simulated sunlight irradiation. These results also indicate that a suitable amount of CaIn_2_S_4_ can effectively improve the photocatalytic performance of ZnIn_2_S_4_ toward the Cr(VI) reduction.

### 3.5. Catalytic Stability

**Figure 7:**
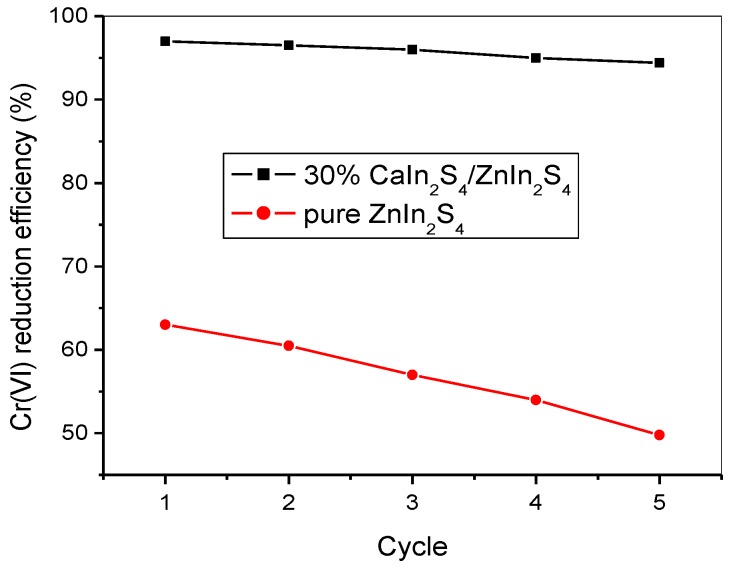
Photocatalytic stability tests of ZnIn_2_S_4_ and 30% CaIn_2_S_4_/ZnIn_2_S_4_ toward the Cr(VI) reduction.

The stability of a given photocatalyst was also an important factor in the practical application [40]. Furthermore, narrow bandgap semiconductors are generally more unstable when exposed to the sunlight illumination [24]. To study the effect of the CaIn_2_S_4_/ZnIn_2_S_4_ heterostructure on the catalytic stability, the cycle experiments of the Cr(VI) reduction were carried out and compared by using ZnIn_2_S_4_ and 30% CaIn_2_S_4_/ZnIn_2_S_4_ as photocatalysts under simulated sunlight irradiation (Figure 7). The results indicate that for the pristine ZnIn_2_S_4_, the reduction efficiency of Cr(VI) decreased about 20.9% after five repeated uses, demonstrating that ZnIn_2_S_4_ was unstable to some extent under simulated sunlight illumination. In comparison, a relatively low decrease in the reduction efficiency of Cr(VI) over 30% CaIn_2_S_4_/ZnIn_2_S_4_ was observed, and only a ca. 2.6% decrease of the reduction efficiency is obtained after reusing five cycles. This suggests that the sunlight stability of ZnIn_2_S_4_ can be improved through forming the heterojunction composites with CaIn_2_S_4_. Additionally, the XPS analysis of the used 30% CaIn_2_S_4_/ZnIn_2_S_4_ catalyst was also performed, and the obtained results are displayed in Figure S2 of the Supplementary Materials. As can be found from Figure S2, the surface element composition and the chemical state of the 30% CaIn_2_S_4_/ZnIn_2_S_4_ sample before and after the photocatalytic reaction show no obvious difference. The results confirm the superior stability of the CaIn_2_S_4_/ZnIn_2_S_4_ composite photocatalysts under simulated sunlight, which is more promising for the practical photocatalytic applications in environmental restoration.

### 3.6. Enhancement Mechanism of Photocatalytic Activity and Stability

**Figure 8:**
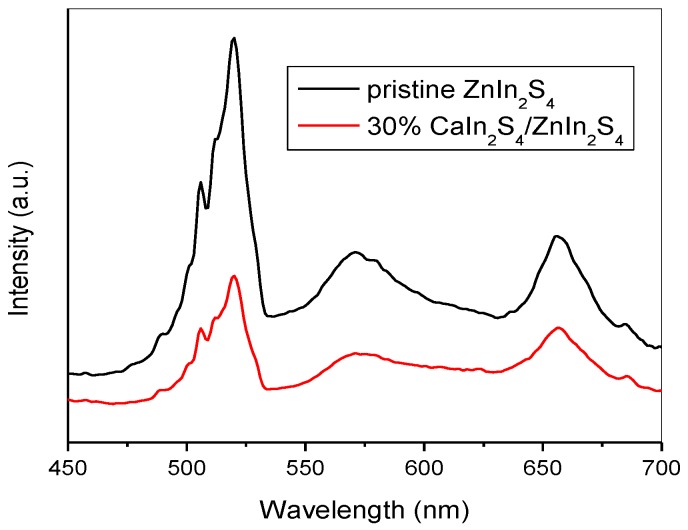
Room temperature photoluminescence spectra (PLs) of the pure ZnIn_2_S_4_ and 30% CaIn_2_S_4_/ZnIn_2_S_4_ under the excitation wavelength of 330 nm.

On the basis of the above discussion and results, it is obvious that coupling a suitable amount of CaIn_2_S_4_ can dramatically improve the photocatalytic performance for the Cr(VI) reduction, including the reduction efficiency and cycling stability of the CaIn_2_S_4_/ZnIn_2_S_4_ composites. The remarkably enhanced photocatalytic activity of the CaIn_2_S_4_/ZnIn_2_S_4_ samples, compared with the pure CaIn_2_S_4_ and ZnIn_2_S_4_, can be ascribed to the effective separation of the photogenerated electron/hole pairs due to the forming of flower-like heterostructures between ZnIn_2_S_4_ and CaIn_2_S_4_. Generally, the photoluminescence spectrum (PLs) is considered as a vital technology to study the migration and fate of photoinduced charge carriers [39,41]. Higher intensities of PL signals usually represent higher recombination rates of photogenerated charge carriers, thus resulting in a lower photocatalytic performance. The comparison of the PL spectrum for the pristine ZnIn_2_S_4_ and 30% CaIn_2_S_4_/ZnIn_2_S_4_ with an excitation wavelength of 330 nm is presented in Figure 8. The pristine ZnIn_2_S_4_ showed emissions at 520, 571, and 656 nm. The strong emission peak centered at 520 nm for the pristine ZnIn_2_S_4_ was attributed to the intrinsic luminescence of ZnIn_2_S_4_. The relatively weak PL peaks at 571, and 656 nm can be ascribed to the surface state emissions, which were mainly caused by the surface defects in the ZnIn_2_S_4_ structure. In comparison with the pristine ZnIn_2_S_4_, there was no new emission signal in the PL spectrum of 30% CaIn_2_S_4_/ZnIn_2_S_4_, but the intensities of the PL peaks decreased obviously. The results imply that the photogenerated electrons and holes can transfer effectively between CaIn_2_S_4_ and ZnIn_2_S_4_, thus suppressing the recombination of charge carriers. This can be a primary reason for the CaIn_2_S_4_/ZnIn_2_S_4_ composites possessing excellent photocatalytic reduction performance under simulated sunlight irradiation.

**Figure 9:**
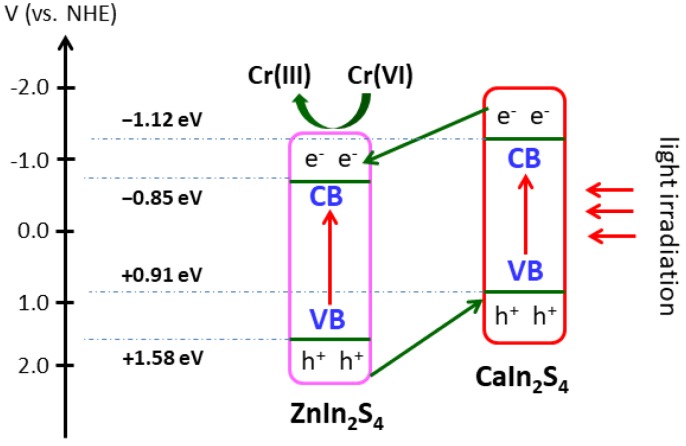
Schematic diagram of the transfer and separation of photogenerated charges in the CaIn_2_S_4_/ZnIn_2_S_4_ composites under simulated sunlight irradiation.

Most of the heterostructured nanocomposites follow the bidirectional charge transfer mechanism [42,43]. For instance, Kumar and his colleagues have reported on the photoinduced electrons enriched on the conduction band of Ag_3_PO_4_ and holes on the valence band of g-C_3_N_4_, which was conducted through a bidirectional charge transfer process between Ag_3_PO_4_ and g-C_3_N_4_ [42]. As for the as-prepared CaIn_2_S_4_/ZnIn_2_S_4_ composites, the aforementioned bidirectional charge transfer was also the primary charge migration process. Based on the bandgap energies of CaIn_2_S_4_ and ZnIn_2_S_4_ estimated from Figure 5 and Equations (2) and (3), the energy band structure diagram of CaIn_2_S_4_ and ZnIn_2_S_4_ can be schematically illustrated, as shown in Figure 9. Under light illumination, the components of CaIn_2_S_4_ and ZnIn_2_S_4_ were simultaneously excited, generating photoinduced electron/hole pairs. Due to the more negative conduction-band edge of CaIn_2_S_4_ (−1.12 eV) than that of ZnIn_2_S_4_ (−0.85 eV), the photogenerated electrons prefer to transfer from the CB of CaIn_2_S_4_ to ZnIn_2_S_4_, whereas the photogenerated holes on the more positive VB of ZnIn_2_S_4_ (+1.58 eV) would migrate to that of CaIn_2_S_4_ (+0.91 eV). This bidirectional charge transfer process results in efficient separation of photogenerated electron/hole pairs in the synthesized CaIn_2_S_4_/ZnIn_2_S_4_ composites.

**Figure 10:**
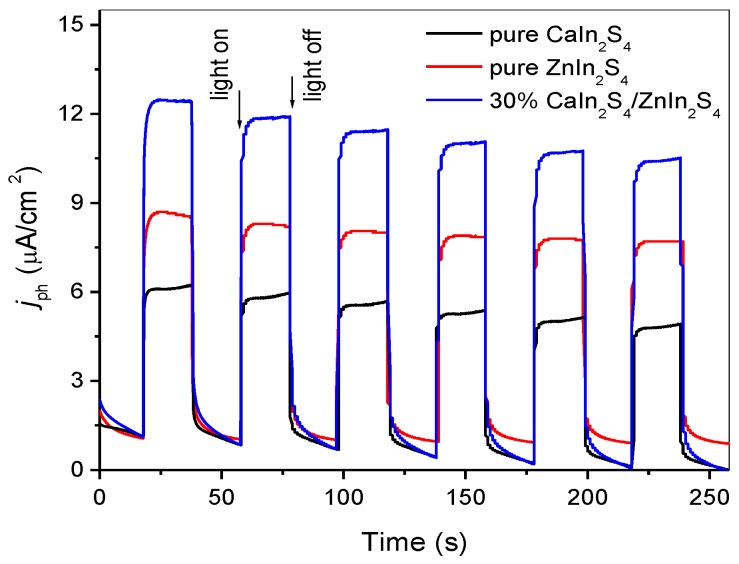
Photocurrent spectra of the as-synthesized pure CaIn_2_S_4_, ZnIn_2_S_4_, and 30% CaIn_2_S_4_/ZnIn_2_S_4_ samples under simulated sunlight irradiation with 20 s light on/off cycles.

To further investigate the important function of heterostructures in enhancing the separation of charge carriers, the transient photocurrent responses were measured over working electrodes made of the pure CaIn_2_S_4_, ZnIn_2_S_4_, and 30% CaIn_2_S_4_/ZnIn_2_S_4_ composite. As depicted in Figure 10, the fast and steady photocurrent response can be detected for each light-on and light-off cycle over the pure CaIn_2_S_4_, ZnIn_2_S_4_, and 30% CaIn_2_S_4_/ZnIn_2_S_4_ composite. The pristine CaIn_2_S_4_ exhibited a very low photocurrent density, whereas the pure ZnIn_2_S_4_ showed a relatively higher photocurrent than that of CaIn_2_S_4_ under simulated sunlight irradiation. This could be due to the fact that CaIn_2_S_4_ possesses a narrower bandgap than ZnIn_2_S_4_, which is unfavorable for the effective separation of photoinduced electron/hole pairs. This would result in the short survival time of photogenerated electrons and weak photocurrent density. However, the composite sample of 30% CaIn_2_S_4_/ZnIn_2_S_4_ exhibits a dramatically enhanced photocurrent density compared with that of the pure ZnIn_2_S_4_ and CaIn_2_S_4_, which further substantiates the efficient separation of the photoinduced electron/hole pairs in the obtained CaIn_2_S_4_/ZnIn_2_S_4_ composites.

Based on the above experimental results and the charge transfer process depicted in Figure 9, a possible enhancement mechanism of photocatalytic activity and stability for the Cr(VI) reduction can be proposed. Under simulated sunlight irradiation, the components of CaIn_2_S_4_ and ZnIn_2_S_4_ are excited to produce photoinduced holes and electrons. Owing to the relatively high recombination rate of photogenerated electron/hole pairs, the pristine CaIn_2_S_4_ and ZnIn_2_S_4_ exhibited low photocatalytic performance. Due to the well-matched energy band structures and the intimate interfacial contact between CaIn_2_S_4_ and ZnIn_2_S_4_ in the as-synthesized composites, the photoinduced electrons located on the CB of CaIn_2_S_4_ can easily migrate to that of ZnIn_2_S_4_, and on the contrary, the photoinduced holes on the VB of ZnIn_2_S_4_ spontaneously transfer to that of CaIn_2_S_4_ (Figure 9). These photoelectrons (e^−^) accumulated on the CB of ZnIn_2_S_4_ possess a strong reduction ability (−0.85 eV vs. NHE (normal hydrogen electrode)), which can reduce toxic Cr(VI) to Cr(III) (*E*_Cr(VI)/Cr(III)_ = +0.55 eV vs. NHE) [44,45]. The photoholes located on the VB of CaIn_2_S_4_ react with the sacrificial reagents immediately. The heterostructured composites formed between CaIn_2_S_4_ and ZnIn_2_S_4_ effectively prevent the recombination of photogenerated electrons and holes, and thus, the photocatalytic activities of the Cr(VI) reduction are enhanced greatly. Meanwhile, the effective transfer of photogenerated holes from the VB of ZnIn_2_S_4_ to that of CaIn_2_S_4_ is beneficial for preventing the oxidation of S^2−^ by holes, which significantly improves the photostability of ZnIn_2_S_4_ in the composite catalysts.

## 4. Conclusions

In short, the CaIn_2_S_4_/ZnIn_2_S_4_ composite photocatalysts were successfully prepared through a one-step hydrothermal process. XRD patterns show that the as-synthesized flower-like composites consist of hexagonal phase ZnIn_2_S_4_ and cubic phase CaIn_2_S_4_. Compared with the pristine ZnIn_2_S_4_, the heterostructured composites CaIn_2_S_4_/ZnIn_2_S_4_ show significantly improved photocatalytic activity and stability for the Cr(VI) reduction under simulated sunlight illumination. The molar content of CaIn_2_S_4_ has a great influence on the photocatalytic activity of the CaIn_2_S_4_/ZnIn_2_S_4_ composites, and 30% CaIn_2_S_4_/ZnIn_2_S_4_ exhibits the optimal photocatalytic performance for the Cr(VI) reduction. A possible mechanism of the photogenerated charge transfer was proposed to illustrate the superior photocatalytic performance and photostability of the CaIn_2_S_4_/ZnIn_2_S_4_ composite catalysts. This study is of great importance in the design and synthesis of heterostructured sulfide composites with excellent photocatalytic performance and consistent stability toward the elimination of toxic metal ions in water.

## Figures and Tables

**Table 1:** BET (Brunauer-Emmett-Teller) specific surface area, molar ratios of Ca–Zn in the synthesized CaIn_2_S_4_/ZnIn_2_S_4_ and apparent quantum efficiency (AQE) of the Cr(VI) reduction over these composites.

Samples	S_BET_ (m^2^/g)	Molar Ratios of Ca:Zn (%)	AQE (%)
pure ZnIn_2_S_4_	59.2	0	3.7
5% CaIn_2_S_4_/ZnIn_2_S_4_	56.3	5.05	4.1
10% CaIn_2_S_4_/ZnIn_2_S_4_	54.7	9.67	4.5
20% CaIn_2_S_4_/ZnIn_2_S_4_	53.4	18.30	5.2
30% CaIn_2_S_4_/ZnIn_2_S_4_	52.1	26.52	6.6
50% CaIn_2_S_4_/ZnIn_2_S_4_	50.5	43.29	5.5
pure CaIn_2_S_4_	46.0	–	2.3

## Supplementary Materials

The following are available online at http://www.mdpi.com/2079-4991/8/7/472/s1, Figure S1: Concentrations of Cr(VI) and total Cr ions in the photocatalytic reaction solution over 30% CaIn2S4/ZnIn2S4 catalyst under simulated sunlight irradiation., Figure S2: XPS survey spectra (a), high-resolution XPS spectra of Zn 2p (b), In 3d (c), Ca 2p (d), and S 2p (e) of 30% CaIn2S4/ZnIn2S4 composite sample before and after the photocatalytic reaction, respectively.
