# Supplementary material for: Facile Synthesis of Novel CaIn2S4/ZnIn2S4 Composites with Efficient Performance for Photocatalytic Reduction of Cr(VI) under Simulated Sunlight Irradiation
Source: Nanomaterials (Basel). 2018 Jun 27;8(7):472. doi: 10.3390/nano8070472 (PMC6071186; doi:10.3390/nano8070472)
Supplement: Supplementary file 1 [file nanomaterials-08-00472-s001.pdf]

Supplementary Materials

Facile synthesis of novel $CaIn_2S_4/ZnIn_2S_4$ composites with efficient performance for photocatalytic reduction of Cr(VI) under simulated sunlight irradiation

Siyu Xu[1], Jun Dai[1,3], Juan Yang[1,2,3*], Jun You[2] and Jingyi Hao[2]

[1] Institute of Chemical Safety, School of Safety Science and Engineering, Henan Polytechnic University, Jiaozuo 454003, P.R. China; xusiyu2017@163.com; daijun@hpu.edu.cn

[2] Institute of Applied Chemistry, College of Chemistry and Chemical Engineering, Henan Polytechnic University, Jiaozuo 454003, P.R. China; youjunhpu@163.com; haojingyi2018@163.com

[3] The Collaborative Innovation Center of Coal Safety Production of Henan, Henan Polytechnic University, Jiaozuo 454003, P.R. China

* Correspondence: yangjuanhpu@163.com; Tel.: +86-391-398-7881

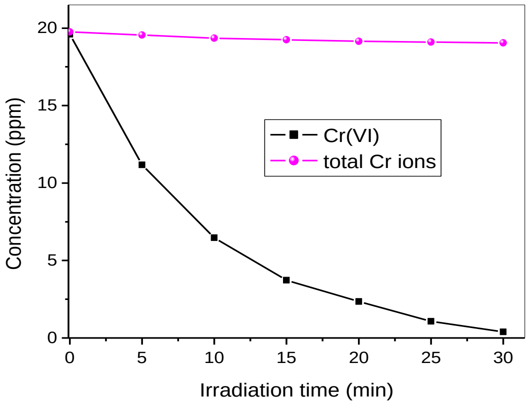

Figure S1. Concentrations of Cr(VI) and total Cr ions in the photocatalytic reaction solution over 30% $CaIn_2S_4/ZnIn_2S_4$ catalyst under simulated sunlight irradiation.

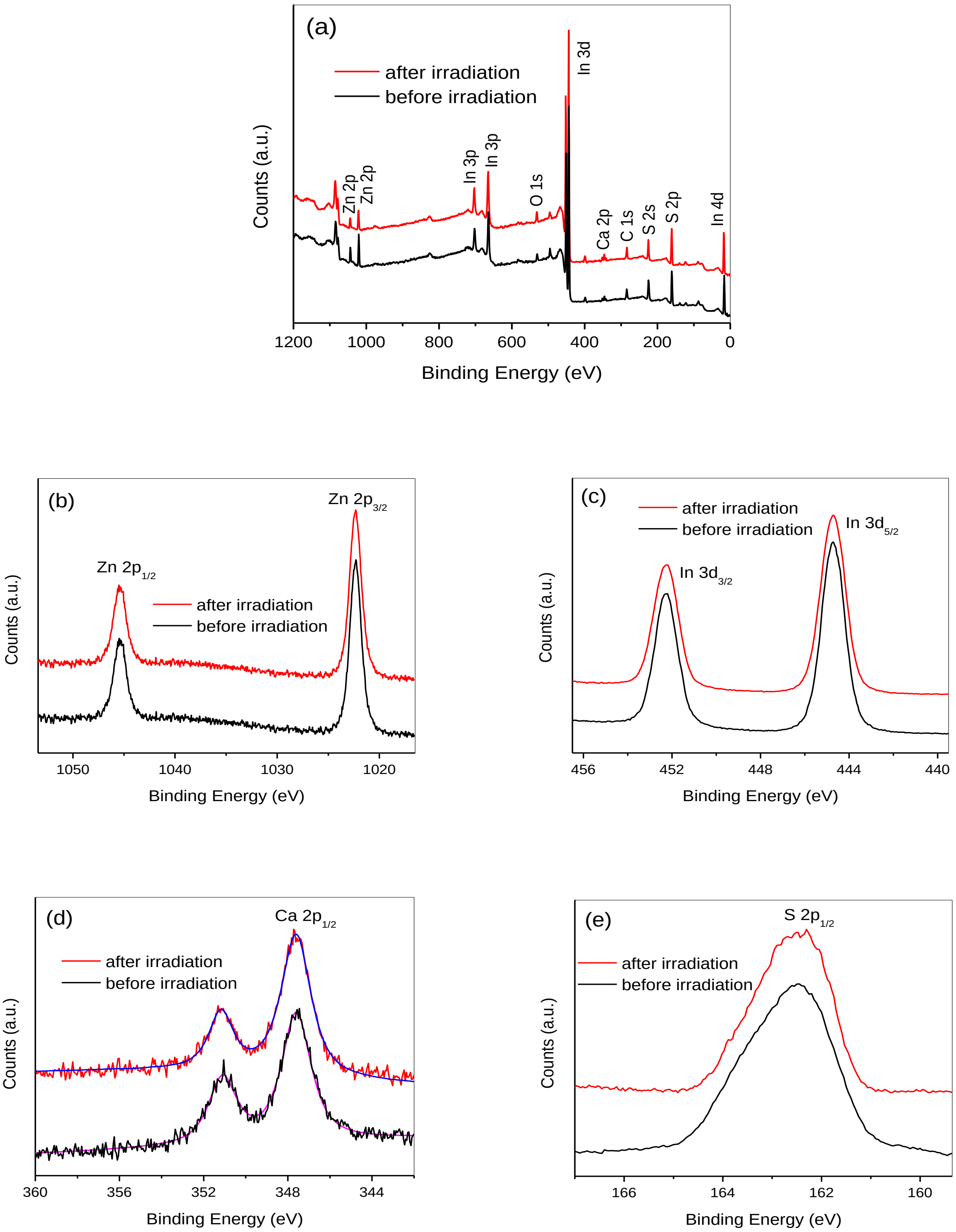

Figure S2. XPS survey spectra (a), high-resolution XPS spectra of Zn 2p (b), In 3d (c), Ca 2p (d), and S 2p (e) of 30% $CaIn_2S_4/ZnIn_2S_4$ composite sample before and after the photocatalytic reaction, respectively.
